# A dose-response relationship between low-density lipoprotein cholesterol levels within the normal range and the incidence of diabetes mellitus: a retrospective cohort study

**DOI:** 10.3389/fendo.2026.1765884

**Published:** 2026-01-28

**Authors:** Yuanshu Tian, Jingjing Tong, Zhe Xue, Xiaolin Lou, Rubing Guo, Wei Zhao

**Affiliations:** 1 School of Public Health, Gansu University of Chinese Medicine, Lanzhou, China; 2 Department of Clinical Laboratory, China-Japan Friendship Hospital, Beijing, China; 3 Department of Infectious Diseases, China-Japan Friendship Hospital, Beijing, China

**Keywords:** cohort study, diabetes mellitus, dyslipidemia, LDL-C, risk factor

## Abstract

**Introduction:**

Despite the established link between elevated Low-density lipoprotein cholesterol (LDL-C) and diabetes, the association of LDL-C levels within the normal range with diabetes is poorly characterized. This study aimed to investigate the relationship between normal-range LDL-C levels and the incidence of new-onset diabetes.

**Methods:**

In this retrospective cohort study, a total of 98,857 individuals with normal LDL-C levels (≤3.4 mmol/L) were enrolled between 2010 and 2016. The association was assessed using Cox proportional hazards regression models. Restricted cubic splines were utilized to examine dose-response relationships and potential nonlinearity. Subgroup analyses were pre-specified by age, sex, and Body mass index (BMI).

**Results:**

During a median follow-up of 3.1 years, 2,107 incident diabetes cases were documented. After multivariable adjustment, participants in the highest level of LDL-C (G4: 2.6–3.4 mmol/L) had a significantly increased risk of diabetes compared to those in the lowest level (G1: 0–1.4 mmol/L), with an adjusted hazard ratios (HR) of 2.11 [95% confidence interval (CI): 1.32–3.38, P = 0.002]. A clear dose-response relationship was observed. Notably, the risk of incident diabetes increased significantly once LDL-C levels exceeded approximately 1.8 mmol/L (Adjusted HR = 1.93, 95% CI: 1.64-2.28, P<0.001). This association remained consistent across all pre-specified subgroups.

**Discussion:**

Among individuals with normal LDL-C levels, higher LDL-C is an independent, dose-dependent risk factor for new-onset diabetes. Maintaining LDL-C below a threshold of approximately 1.8 mmol/L may be associated with a lower diabetes risk, suggesting its potential role in refining primary prevention strategies.

## 1 Introduction

Diabetes mellitus represents a major global public health challenge, affecting approximately 8.5% of the adult population worldwide. Its prevalence, however, is not uniform and rises sharply with age, reaching a disproportionate 15.7% among adults aged 65–80 years (1, 2). Notably, the onset of diabetes is occurring earlier, with studies indicating it now presents an average of 14.6 years prematurely (3). Compounding this issue, the decade from 2007 onwards witnessed a steady increase in diabetes prevalence among Chinese adults—a concerning trend that has persisted in recent years (4).

The high prevalence of type 2 diabetes in China is frequently accompanied by clinically significant dyslipidemia, a well-recognized component of the disease characterized by elevated LDL-C, low high-density lipoprotein cholesterol (HDL-C), and a preponderance of small, dense low-density lipoprotein (sd-LDL) particles (5–7). Consequently, LDL-C lowering, primarily with statins and potentially augmented by non-statin agents, forms the cornerstone of cardiovascular risk management in these patients (6) (8–12).

A substantial body of evidence, including prospective cohort and genetic studies, generally supports a positive association between elevated LDL-C levels and an increased risk of incident type 2 diabetes (13–15). However, this established paradigm is challenged by several intriguing observations reporting an inverse relationship, particularly in statin-naïve individuals, where lower baseline LDL-C was linked to a higher diabetes risk (16–19). This paradoxical finding suggests a more complex role of LDL-C in diabetes pathogenesis than previously understood and highlights the potential value of baseline LDL-C in risk stratification.

Our previous work demonstrated that baseline levels of triglycerides and the Triglyceride-glucose Index (TyG) index, even at levels considered within the reference range, were longitudinally associated with an elevated future risk of diabetes (20, 21). While the association between elevated LDL-C and diabetes is established, the specific association between LDL-C levels within the normal range and new-onset diabetes remains poorly defined, particularly in the Chinese population. In this study, we aimed to elucidate the association and dose-response relationship between normal-range LDL-C levels and diabetes risk using data from the China Health and Retirement Longitudinal Study (CHARLS).

## 2 Methods

### 2.1 Data sources and study population

**Figure 1:**
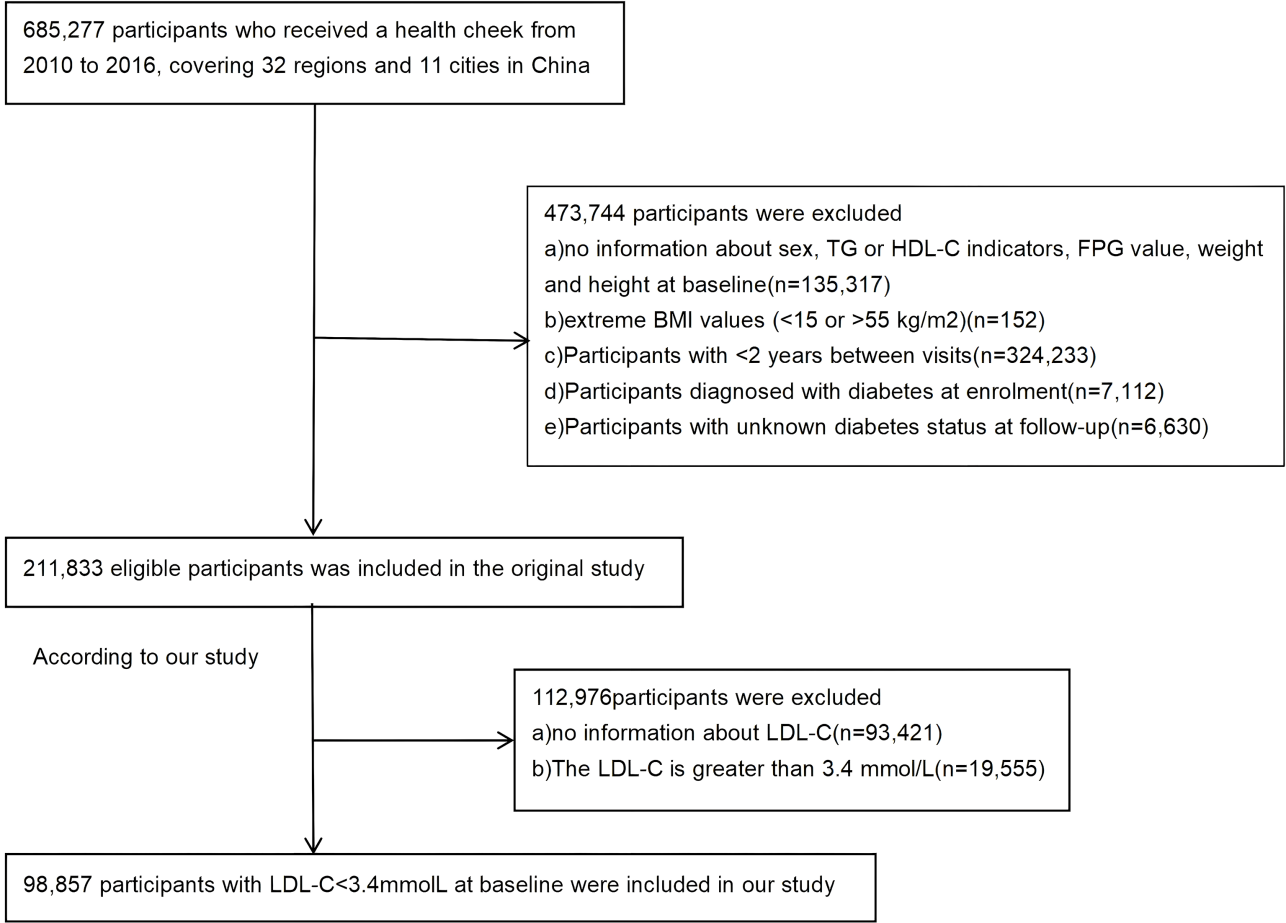
Flow diagram of subjects included in the cohort study.

The primary data for this study were derived from the de-identified electronic health records maintained by the China Rich Healthcare Group. (www.datadryad.org). To complement our primary dataset, we incorporated data from a previously published investigation into the relationships among BMI, age, and diabetes incidence in Chinese adults (Dryad, https://doi.org/10.5061/dryad.ft8750v). The study utilized medical examination records from health screening programs conducted at 32 research centers across 11 major Chinese cities between 2010 and 2016. The initial cohort comprised 685,277 participants aged over 20. Baseline data were derived from the participants’ first examination. Each participant had at least two follow-up visits spaced at least two years apart, with a median follow-up of 3.1 years. The study endpoint was defined as the date of diabetes diagnosis or the last follow-up visit, whichever occurred first. After applying exclusion criteria consistent with a prior publication, the final analytical sample included 211,833 individuals (20–22). The application of additional exclusion criteria, guided by the AACE Clinical Practice Guidelines, resulted in the exclusion of 112,976 participants for missing baseline LDL-C or levels ≥3.4 mmol/L. Consequently, the final analytic cohort comprised 98,857 individuals with baseline LDL-C below 3.4 mmol/L (**Figure 1**).

### 2.2 Data collection

Age and sex were determined using official identification documents. Demographic and lifestyle data, including smoking status, alcohol use, and family history of diabetes, were assessed via structured interviews. Anthropometric measurements [height, weight, BMI, and systolic and diastolic blood pressure (SBP and DBP)] were conducted according to the standardized protocols described previously. Following an overnight fast (≥8 hours), venous blood was drawn and analyzed on an automated biochemistry analyzer for concentrations of fasting plasma glucose (FPG), LDL-C, cholesterol, triglycerides, HDL-C, alanine aminotransferase (ALT), aspartate aminotransferase (AST), and blood urea nitrogen (BUN). Participants were stratified into four groups (G1–G4) based on their baseline LDL-C levels (≤1.4, 1.4–1.8, 1.8–2.6, and 2.6–3.4 mmol/L), in accordance with the clinically relevant thresholds outlined in the ESC/EAS Guidelines for the Management of Dyslipidemia. At all follow-up visits, FPG was remeasured to track longitudinal changes.

### 2.3 Definitions

The diagnosis of incident diabetes was established based on the World Health Organization criterion of a FPG concentration≥7.0 mmol/L or self-reported diabetes during the follow-up period.

### 2.4 Data analysis

All statistical analyses were performed using R (version 4.2.0) and EmpowerStats. A two-sided p-value < 0.05 defined statistical significance. Baseline characteristics were summarized for participants stratified by the four predefined LDL-C groups, presented as mean ± standard deviation for normally distributed continuous variables, median (interquartile range) for non-normal variables, and number (percentage) for categorical variables. The association between LDL-C levels and incident diabetes was evaluated using Cox proportional hazards regression across three models: a crude model; Model I, adjusted for age, sex, HDL-C, FPG, cholesterol, and triglycerides; and Model II, which further incorporated BMI, SBP, DBP, and family history of diabetes. Results are reported as HRs with 95% CIs. Kaplan-Meier curves visualized diabetes-free survival across LDL-C strata. The dose-response relationship was assessed with restricted cubic splines. Subgroup analyses were conducted by sex, BMI, and age. Threshold effects were examined using likelihood ratio tests (LRT), with a p-value < 0.05 indicating significant nonlinearity.

## 3 Results

### 3.1 Individual baseline characteristics

This study analyzed a cohort of 98,857 individuals with LDL-C levels within the normal range. The mean age was 43.2 years, and 53.7% of participants were male. As detailed in **Table 1**, baseline characteristics were stratified by LDL-C concentration thresholds (1.4, 1.8, 2.6, and 3.4 mmol/L). Trends across these strata revealed that participants with higher LDL-C levels were more likely to be male, older, current smokers, and alcohol consumers, and exhibited elevated measures of BMI, SBP, DBP, FPG, ALT, HDL-C, and BUN. Except for family history of diabetes, all variables demonstrated statistically significant differences (P ≤ 0.001).

**Table 1:** Baseline characteristics of participants (N = 98857).

LDL-C(mmol/L) categorical	G1(0-1.4)(n=1031)	G2(1.4-1.8)(n=5018)	G3(1.8-2.6) (n=46291)	G4(2.6-3.4) (n=46517)	P-value
Age (y)	40.3 ± 12.1	39.4 ± 11.8	41.6 ± 12.2	45.3 ± 13.0	<0.001
BMI (kg/m2)	22.7 ± 3.4	22.3 ± 3.2	22.8 ± 3.2	23.7 ± 3.3	<0.001
SBP (mmHg)	116.9 ± 16.8	115.3 ± 15.3	117.4 ± 15.9	120.4 ± 16.8	<0.001
DBP (mmHg)	72.2 ± 11.3	71.6 ± 10.3	73.1 ± 10.7	75.2 ± 11.0	<0.001
FPG (mmol/L)	4.8 ± 0.7	4.9 ± 0.6	4.9 ± 0.6	5.0 ± 0.6	<0.001
Cholesterol (mmol/L)	3.3 ± 1.0	3.5 ± 0.5	4.2 ± 0.5	5.0 ± 0.5	<0.001
Triglyceride (mmol/L)	0.9 (0.6-1.6)	0.8 (0.6-1.3)	0.9 (0.7-1.4)	1.2 (0.8-1.7)	<0.001
HDL-C (mmol/L)	1.2 ± 0.4	1.3 ± 0.3	1.3 ± 0.3	1.4 ± 0.3	<0.001
LDL-C(mmol/L)	1.2 ± 0.2	1.7 ± 0.1	2.3 ± 0.2	3.0 ± 0.2	<0.001
ALT(U/L)	17.0(12.0-26.7)	15.9(11.8-23.0)	16.7(12.0-25.0)	19.0(13.5-28.5)	<0.001
AST(U/L)	21.5(18.0-26.0)	20.9(17.3-25.8)	21.0(18.0-25.6)	22.5(19.0-27.0)	<0.001
BUN (mmol/L)	4.4 ± 1.2	4.4 ± 1.2	4.6 ± 1.2	4.8 ± 1.2	<0.001
Gender, n (%)					<0.001
Male	571 (55.4)	2508 (50.0)	23808 (51.4)	26211 (56.3)	
Female	460 (44.6)	2510 (50.0)	22483 (48.6)	20306 (43.7)	
smoking status, n (%)					<0.001
current smoker	53 (13.0)	241 (14.0)	2335 (17.9)	2855 (22.1)	
ever smoker	7 (1.7)	62 (3.6)	532 (4.1)	534 (4.1)	
never smoker	349 (85.3)	1413 (82.3)	10211 (78.1)	9547 (73.8)	
drinking status, n (%)					<0.001
current drinker	10 (2.4)	44 (2.6)	314 (2.4)	403 (3.1)	
ever drinker	44 (10.8)	261 (15.2)	2186 (16.7)	2228 (17.2)	
never drinker	355 (86.8)	1411 (82.2)	10578 (80.9)	10305 (79.7)	
Family history of diabetes, n (%)					0.723
No	1003(97.28)	4904(97.73)	45235(97.72)	45482(97.78)	
Yes	28 (2.72)	114 (2.27)	1056 (2.28)	1035 (2.22)	

Continuous variables with a normal distribution were reported as mean ± standard deviation (SD), whereas skewed continuous variables were presented as medians (interquartile range [IQR]). Categorical data are presented as n (%). To assess the presence of statistically significant differences among the quartiles, the chi-square test was employed for categorical variables, the Kruskal-Wallis H test was used for skewed variables, and one-way analysis of variance (ANOVA) was applied for variables with a normal distribution. BMI, Body Mass Index; SBP, Systolic Blood Pressure; DBP, Diastolic Blood Pressure; HDL-C, High-Density Lipoprotein Cholesterol; FPG, Fasting Plasma Glucose; LDL-C, Low-Density Lipoprotein Cholesterol; AST, Aspartate Aminotransferase; BUN, Blood Urea Nitrogen; ALT, Alanine Aminotransferase.

### 3.2 Newly diagnosed diabetes

**Figure 2:**
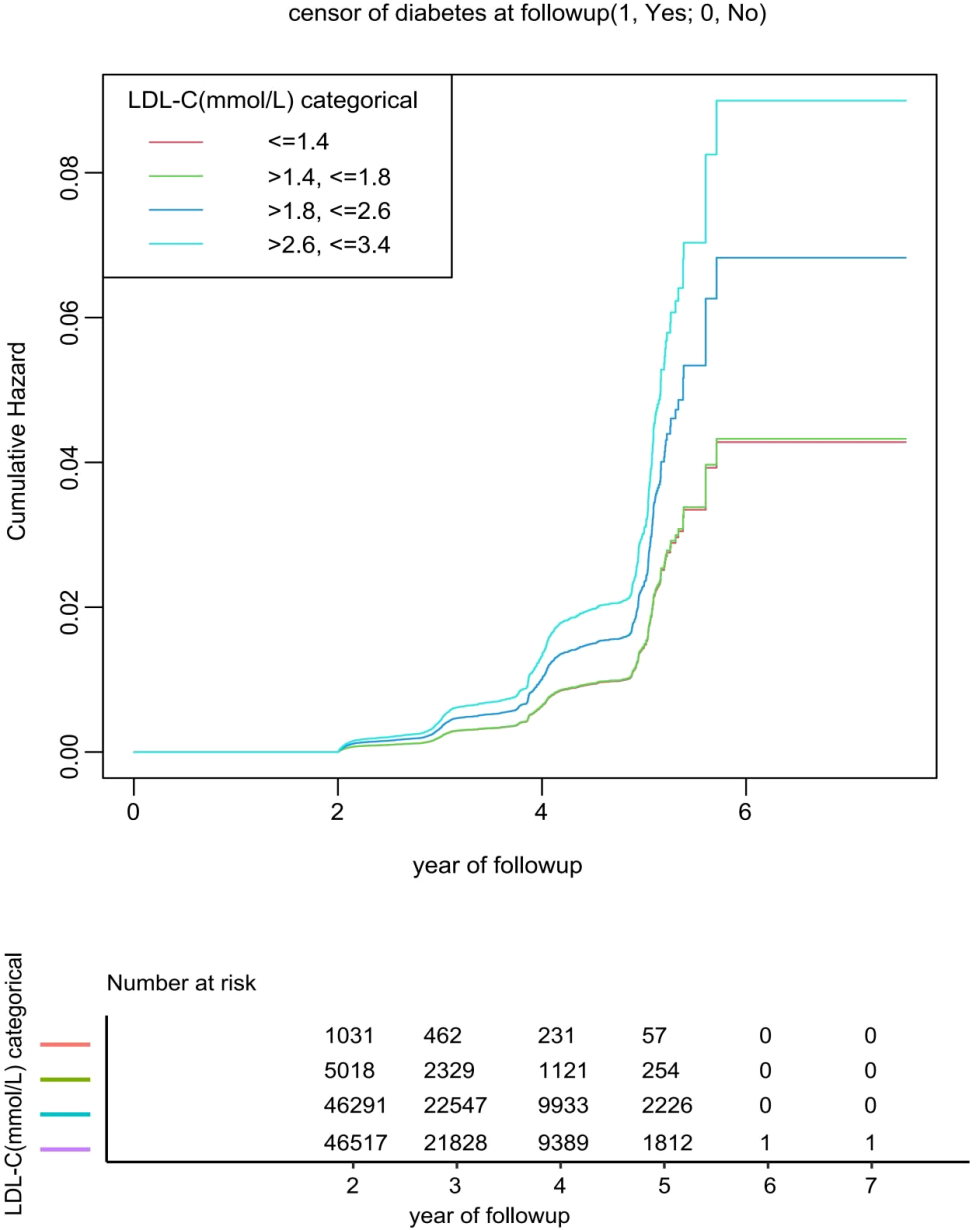
Kaplan-meier analysis of diabetes mellitus type 2 incidence stratified by four cutoff groups for low-density lipoprotein cholesterol levels (log-rank test, P< 0.001). Cumulative Hazard Curve adjustments were made based on Age, Gender, BMI, SBP, DBP, FPG, Cholesterol, Triglyceride, HDL-C, Family history of diabetes. Grouping criteria: G1: ≤1.4 mmol/L, G2: >1.4, ≤1.8 mmol/L, G3:>1.8,≤2.6mmol/L, G4: >2.6, ≤3.4 mmol/L. LDL-C, Low-density lipoprotein cholesterol.

Over a mean follow-up period of 3.1 years, 2,107 out of 98,857 participants (2.13%) developed diabetes. The incidence rates stratified by group were as follows: Group 1 (≤1.4 mmol/L) accounted for 22 cases (2.13%), Group 2 (1.4–1.8 mmol/L) for 81 cases (1.61%), Group 3 (1.8–2.6 mmol/L) for 882 cases (1.91%), and Group 4 (2.6–3.4 mmol/L) for 1,122 cases (2.41%). As illustrated in **Figure 2**. Kaplan-Meier survival analysis demonstrated that participants in Group 4 exhibited the highest cumulative incidence of diabetes, characterized by the most pronounced increase in risk over time, followed by those in Group 3.

### 3.3 Association between normal LDL levels and diabetes incidence

**Table 2** presents the results from a Cox proportional hazards regression analysis that assessed the association of low-density lipoprotein cholesterol with the incidence of diabetes. When LDL-C was analyzed as a continuous variable, each unit increase was associated with an unadjusted HR of 1.43 (95% CI: 1.30–1.57, p < 0.001). After full adjustment in Model II, the effect estimate increased to an HR of 1.90 (95% CI: 1.63, 2.21, p < 0.001). In categorical analyses using the lowest LDL-C group (G1: ≤1.4 mmol/L) as reference, the fully adjusted Model II yielded the following risk estimates: G2 (1.4–1.8 mmol/L), HR = 1.02 (95% CI: 0.63–1.66, p = 0.938); G3 (1.8–2.6 mmol/L), HR = 1.61 (95% CI: 1.02, 2.53, p = 0.040); and G4 (2.6–3.4 mmol/L), HR = 2.11 (95% CI: 1.32–3.38, p = 0.002). A sensitivity analysis excluding self-reported diabetic patients was performed. In the fully adjusted Model II, the corresponding HR was 0.91 (95% CI: 0.54–1.54; p = 0.730) for G2 (1.4–1.8 mmol/L), 1.58 (95% CI: 0.98–2.56; p = 0.063) for G3 (1.8–2.6 mmol/L), and 1.99 (95% CI: 1.20–3.29; p = 0.007) for G4 (2.6–3.4 mmol/L) (**Supplementary Table S1**).

**Table 2:** Relationship between normal-range LDL-C levels and incidence of diabetes mellitus.

Outcome	Crude model		Model I		Model II	
	HR (95% CI)	P-value	HR (95% CI)	P-value	HR (95% CI)	P-value
LDL-C(mmol/L)	1.43(1.30,1.57)	<0.001	2.04(1.75,2.37)	<0.001	1.90(1.63, 2.21)	<0.001
LDL-C(mmol/L) categorical						
<=1.4	Reference		Reference		Reference	
>1.4, <=1.8	0.76(0.47,1.22)	0.255	1.10(0.68,1.77)	0.708	1.02(0.63, 1.66)	0.938
>1.8, <=2.6	0.89(0.58,1.36)	0.589	1.82(1.17,2.85)	0.008	1.61(1.02, 2.53)	0.040
>2.6, <=3.4	1.17(0.77,1.79)	0.466	2.51(1.58,3.99)	<0.001	2.11(1.32, 3.38)	0.002
P for trend	<0.001		<0.001		<0.001	

Model I was adjusted for Age, Gender, FPG, Cholesterol, Triglyceride, HDL-C.

Model II was adjusted for Age, Gender, BMI, SBP, DBP, FPG, Cholesterol, Triglyceride, HDL-C, Family history of diabetes.

HR, hazard ratios; CI, confidence interval; other abbreviations as in **Table 1**.

### 3.4 Threshold effect analysis of LDL-C and diabetes incidence

**Figure 3:**
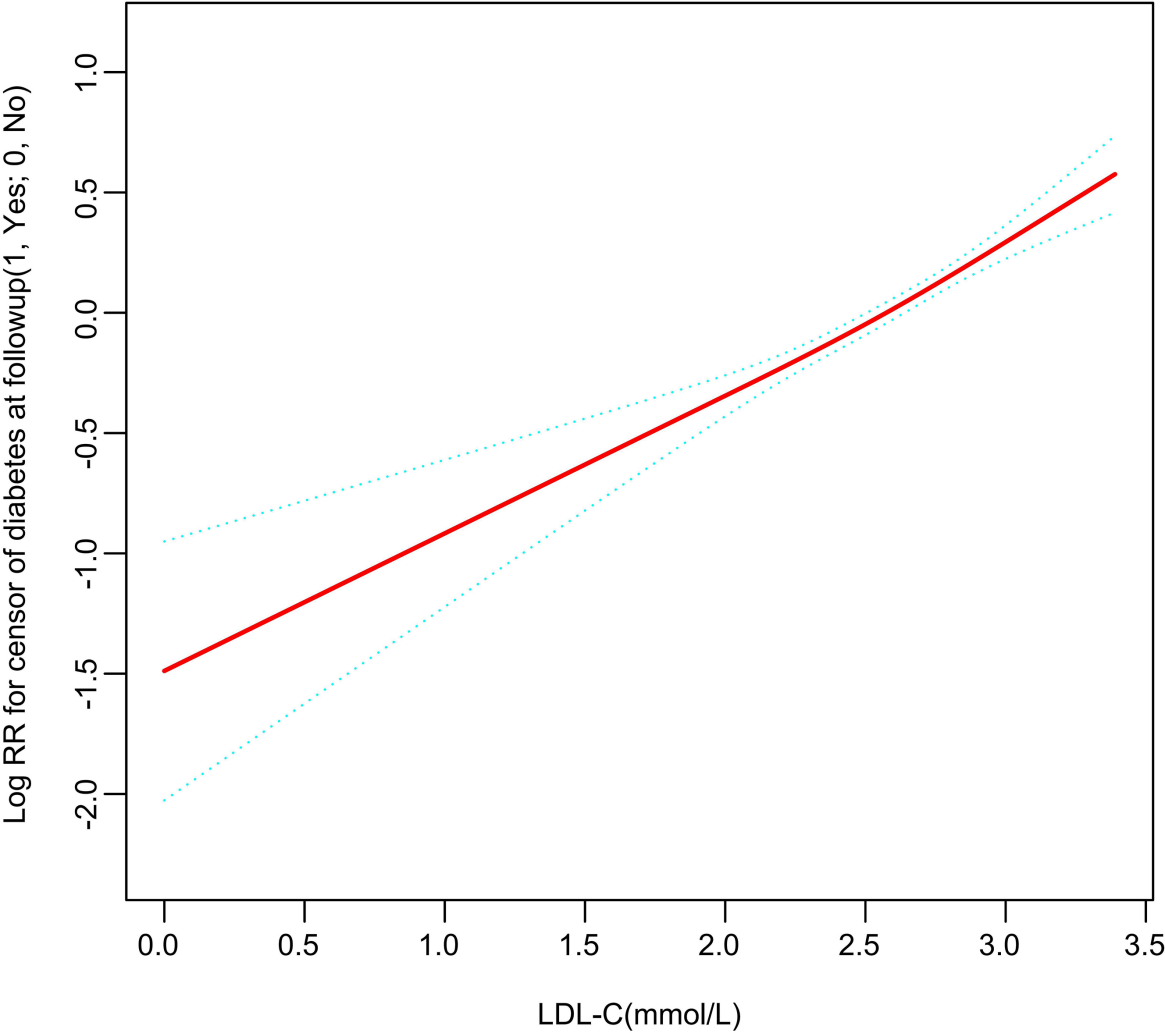
A linear and non-threshold association between LDL-C levels and incident diabetes risk was demonstrated by restricted cubic spline analysis. The solid red line depicts the covariate-adjusted risk of type 2 diabetes mellitus, with the shaded blue area representing the corresponding 95% confidence interval. All adjusted for Age, Gender, BMI, SBP, DBP, FPG, Cholesterol, Triglyceride, HDL-C, and family history of diabetes.

The dose-response relationship between LDL-C within the normal range and diabetes risk was modeled using restricted cubic splines (RCS). After full adjustment, the smoothed curve displayed an overall positive and approximately linear association (**Figure 3**). The likelihood ratio test comparing a linear model to a model with a threshold at 1.8 mmol/L did not show a statistically significant improvement in fit (P = 0.545), indicating insufficient evidence to definitively reject a linear relationship in favor of a threshold model. However, motivated by visual inspection and clinical relevance, we performed an exploratory piecewise linear regression. This analysis suggested an inflection point around 1.8 mmol/L. At LDL-C levels below this point, the association with diabetes risk was not statistically significant (HR = 1.58, 95% CI: 0.87–2.87, P = 0.130). In contrast, for levels above 1.8 mmol/L, each unit increase was associated with a significantly higher risk (HR = 1.93, 95% CI: 1.64–2.28, P < 0.001). Detailed results are provided in **Table 3**.

**Table 3:** Threshold effect analysis of the LDL-C ratio and incidence of diabetes mellitus.

Model	LDL-C increase	
	HR (95%CI)	P value
Model I		
One line effect	1.90 (1.63, 2.21)	<0.001
Model II		
Turning point (K)	1.8	
LDL-C ratio < K	1.58 (0.87,2.87)	0.130
LDL-C ratio > K	1.93 (1.64,2.28)	<0.001
P value for LRT test*		0.545

Adjustments were made based on Age, Gender, BMI, SBP, DBP, FPG, Cholesterol, Triglyceride, HDL-C, Family history of diabetes.

HR, hazard ratios; CI, confidence interval; LRT, Likelihood Ratio; other abbreviations as in **Table 1**.

### 3.5 Subgroup analysis

**Figure 4:**
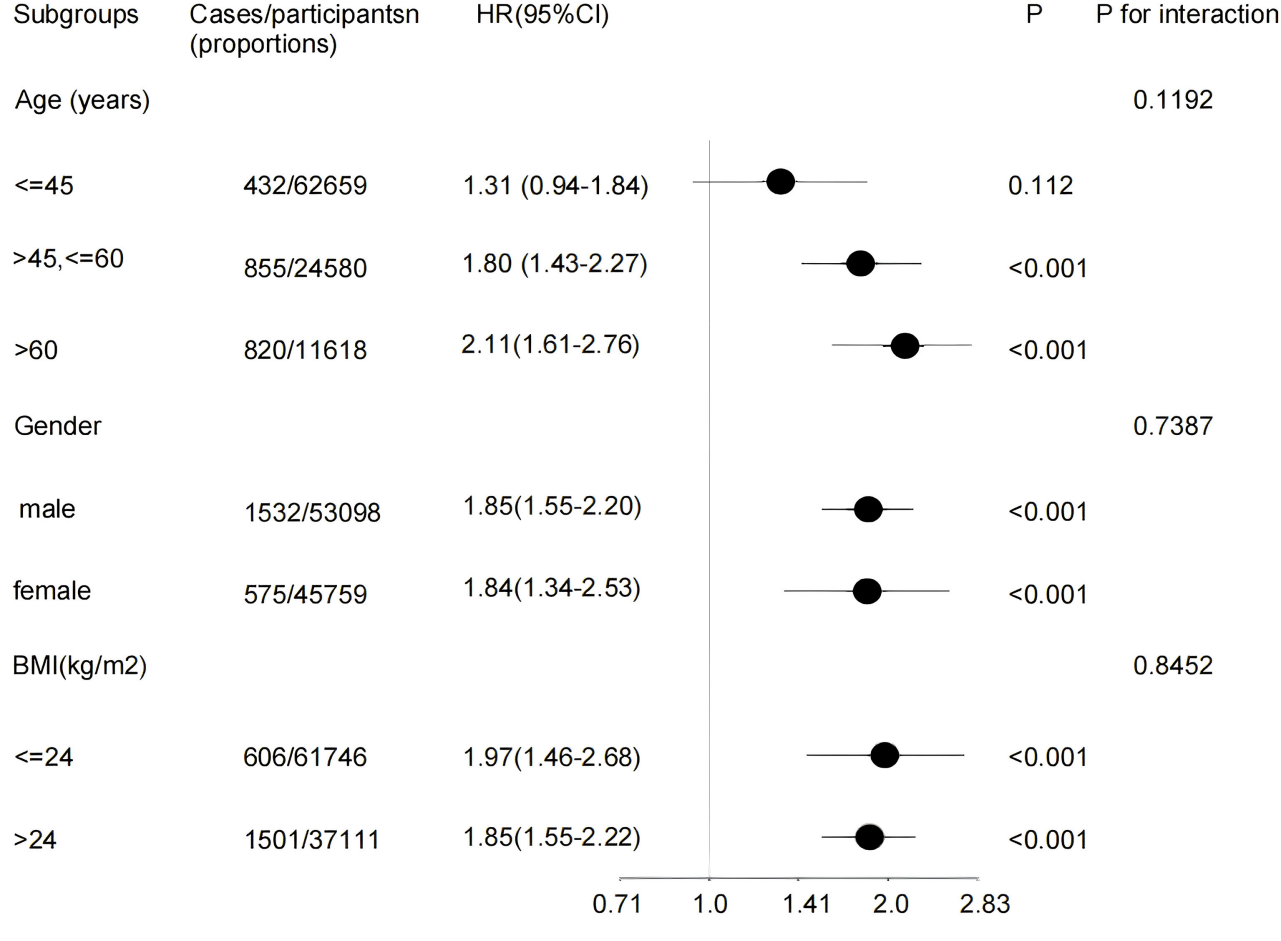
Effect size of LDL-C ratio and incidence of diabetes mellitus in prespecified and exploratory subgroups in each subgroup. Adjustment sets varied by stratification: models included all covariates (age, gender, BMI, SBP, DBP, FPG, Cholesterol,Triglycerides, HDL-C and family history of diabetes) except the variable used for stratification. HR, hazard ratios; CI, confidence interval; BMI, Body Mass Index.

As detailed in **Figure 4**, subgroup analyses confirmed that the positive association between LDL-C levels and diabetes incidence was robust and consistent across all strata of age, sex, and BMI (P for interaction = 0.1192, 0.7387, 0.8452, respectively). This relationship remained evident regardless of these demographic and anthropometric factors.

## 4 Discussion

This retrospective cohort study establishes a positive, dose-dependent association between LDL-C levels within the normal range (<3.4 mmol/L) and incident diabetes among Chinese adults (age ≥20 years). Each 1 mmol/L increment in LDL-C was associated with a 90% increase in diabetes risk. The relationship was best described by a linear model (P for nonlinearity = 0.545), with a notable inflection point at 1.8 mmol/L. Beyond this threshold, the risk escalated markedly (HR = 1.93, P < 0.001). This association remained robust and consistent across all subgroups of age, sex, and BMI.

Insulin resistance and defective insulin secretion, the hallmarks of type 2 diabetes (T2DM), are closely linked to alterations in lipid metabolism (23). Notably, LDL-C, a particle defined by its apolipoprotein B-100 (ApoB-100) component and derived from hepatic synthesis, plays a significant role in this context (24–27). Following its secretion, the precursor lipoprotein undergoes subsequent remodeling mediated by lipoprotein lipase (LPL), cholesteryl ester transfer protein (CETP), and hepatic lipase to form mature LDL (28). From a metabolic perspective, elevated levels of LDL-C are prone to oxidative modification, yielding oxidized LDL (ox-LDL). This ox-LDL stimulates the activation of vascular endothelial and immune cells, thereby initiating a state of chronic inflammation and disrupting insulin signaling pathways. The consequent impairment of insulin sensitivity in peripheral tissues such as muscle and adipose tissue ultimately promotes systemic insulin resistance and can compromise pancreatic beta-cell integrity (29–31). Elevated LDL-C levels are closely associated with obesity and hypertriglyceridemia, core components of metabolic syndrome (32, 33). The expansion of adipose tissue promotes the release of pro-inflammatory adipokines, which exacerbate insulin resistance—a key pathway that further aggravates the risk of diabetes development (34).

Prior research has demonstrated a correlation between initial LDL-C levels and the later onset of diabetes (14, 15, 17, 35–37). For instance, a 2019 analysis employing restricted cubic spline regression delineated a U-shaped relationship between LDL-C and diabetes risk: risk was higher at low LDL-C concentrations, descended to a nadir at moderate levels, and rose once more as LDL-C levels increased (17). A 10-year prospective study within the Isfahan cohort demonstrated that among non-diabetic first-degree relatives (FDRs) of diabetic patients (aged 30-70), elevated LDL-C levels were significantly associated with an increased risk of developing T2DM (HR: 1.22, 95% CI: 1.09–1.36), independent of lipid-lowering therapy at baseline (35). Cross-sectional data indicated a graded relationship between small, dense LDL-C (sdLDL-C) and dysglycemia. Per 0.1 mmol/L increment in sdLDL-C, the odds of prediabetes increased by 3.4% (OR = 1.034, 95% CI: 1.002–1.067), while the odds of newly diagnosed T2DM increased by 15.7% (OR = 1.157, 95% CI: 1.097–1.220), despite non-significant overall associations (36). Mendelian randomization studies have established elevated LDL-C as a causal driver of T2DM, reporting a 25% increased risk per unit rise in LDL-C (beta = 0.25, 95% CI: 0.105–0.395, p = 0.001) (14). Nevertheless, whether LDL-C levels within the normal physiological range similarly confer risk remains incompletely understood. Evidence regarding the diabetogenic risk of LDL-C within the physiological range remains limited. A 2023 community-based Chinese study reported that higher LDL-C levels were associated with an increased incidence of new-onset diabetes (OR: 1.12, 95% CI: 1.03–1.22), with each 1 mmol/L increment below 3.4 mmol/L elevating the risk by 24% (OR: 1.24, 95% CI: 1.02–1.51) (15). This positive association aligns with our findings of a substantially elevated risk (HR: 2.11, 95% CI: 1.32–3.38) in the highest normal-range subgroup, thereby validating the continuous risk associated with physiological LDL-C levels. The present analysis delineated a dose-response relationship between LDL-C within the normal range and diabetes incidence using smooth curve fitting, which yielded an overall linear association. Threshold analysis suggested a potential inflection point at 1.8 mmol/L, although the overall test for nonlinearity was not statistically significant (P > 0.05). Below this level, the HR for diabetes was 1.58 (95% CI: 0.87–2.87, P = 0.130), whereas above 1.8 mmol/L, the risk increased significantly (HR = 1.93, 95% CI: 1.64–2.28; P < 0.001). Categorical Cox regression analysis corroborated this pattern. Compared with the reference group (LDL-C ≤1.4 mmol/L), the risk was not significantly elevated in the 1.4–1.8 mmol/L group (HR = 1.02, 95% CI: 0.63–1.66, P = 0.938). However, the risk was 61% higher in the 1.8–2.6 mmol/L group (HR = 1.61) and 111% higher in the 2.6–3.4 mmol/L group (HR = 2.11). The potential benefit of maintaining LDL-C below 1.8 mmol/L for diabetes prevention, as indicated by our analysis, aligns with existing evidence. A previous investigation similarly demonstrated a significant positive correlation between LDL-C and prediabetes risk after adjustment (HR: 1.49, 95% CI: 1.40–1.58, p < 0.001). Moreover, that study identified a nonlinear association with an inflection point at 2.19 mmol/L (log-likelihood ratio test p < 0.001), further reinforcing the concept of a threshold effect. These convergent findings underscore the relevance of optimal LDL-C levels, even within the normal range, in primary diabetes prevention (37). Furthermore, stratified analyses by age, sex, and BMI further validated this relationship, underscoring the robustness of our findings across key demographic and anthropometric subgroups.

This study possesses several key strengths. Primarily, it addresses a significant gap by employing a large, population-based retrospective cohort to investigate the LDL-C-diabetes relationship. Our study’s major contribution is its detailed dose-response analysis, uncovering a previously unrecognized positive association with a distinct threshold effect within the normal LDL-C range, established through rigorous adjustment for potential confounders. Furthermore, the consistency of this association across all examined subgroups underscores its robustness and generalizability. Despite these strengths, this study has several limitations. First, although no distinction was made between type 1 and type 2 diabetes, the vast majority of cases in adults are type 2 diabetes, which aligns with the primary focus of this investigation (38). Second, LDL-C was measured in the fasting state, where it fulfills essential physiological functions. The robust association observed even under these conditions suggests it is a strong marker of metabolic risk. However, future studies incorporating postprandial lipid measures or lipoprotein particle characteristics could provide further mechanistic insight. Third, the fixed-baseline design precluded the evaluation of longitudinal variations in key confounders such as BMI, lipid profiles, and lifestyle factors. This limitation could introduce residual confounding, as a single baseline assessment may not accurately reflect an individual’s status throughout the follow-up period. Fourth, incident diabetes was defined by fasting plasma glucose (≥7.0 mmol/L) or self-reported diagnosis, without HbA1c or OGTT confirmation. This may have resulted in non-differential misclassification and an underestimation of the true incidence. Notably, a sensitivity analysis excluding self-reported cases yielded consistent results, supporting the robustness of our primary finding despite this methodological limitation. Fifth, data regarding the use of lipid-lowering, glucose-lowering, or other cardiometabolic medications were not available. The absence of this information represents a potential source of unmeasured confounding, given that such therapies can modulate both LDL-C levels and diabetes risk. Finally, this cohort was derived from a voluntary health screening population, resulting in a mean age of approximately 40–45 years. While this distribution is common in such studies, it may limit the precise estimation of risk to younger adults (e.g., those aged 20–39 years). Nevertheless, our age-stratified analysis confirmed the association was consistent across age groups. Furthermore, this study was conducted exclusively in Chinese adults. Given known ethnic differences in lipid profiles and diabetes epidemiology, our findings—particularly the observed association and the potential inflection point at 1.8 mmol/L—require validation in diverse populations before their broader applicability can be established.

## 5 Conclusion

This study establishes a significant positive association between LDL-C levels within the normal range and the incidence of diabetes. Our analysis identified a potential inflection point at 1.8 mmol/L, beyond which the risk of diabetes increased more markedly. These findings underscore the potential utility of monitoring LDL-C, even within its normative range, for stratifying diabetes risk. However, the clinical implications of this specific threshold for diabetes prevention remain to be established. Whether maintaining LDL-C below 1.8 mmol/L confers a preventive benefit requires verification in prospective interventional trials.

## Data Availability

The original contributions presented in the study are included in the article/**Supplementary Material**. Further inquiries can be directed to the corresponding author.
