# Supplementary material for: A dose-response relationship between low-density lipoprotein cholesterol levels within the normal range and the incidence of diabetes mellitus: a retrospective cohort study
Source: Front Endocrinol (Lausanne). 2026 Jan 28;17:1765884. doi: 10.3389/fendo.2026.1765884 (PMC12890619; doi:10.3389/fendo.2026.1765884)
Supplement: Supplementary file 1 [file Table1.docx]

# Supplementary Material

**Supplementary Table S1** Association between normal-range low-density lipoprotein cholesterol levels and the incidence of diabetes after excluding self-reported diabetic patients.

| Outcome | Crude Model | | Model I | | Model II | |
| --- | --- | --- | --- | --- | --- | --- |
|  | HR (95% CI) | P-value | HR (95% CI) | P-value | HR (95% CI) | P-value |
| LDL-C(mmol/L) | 1.39 (1.25, 1.54) | <0.001 | 2.02 (1.72, 2.38) | <0.001 | 1.87 (1.58, 2.20) | <0.001 |
| LDL-C(mmol/L) categorical | | | | | | |
| <=1.4 | Reference |  | Reference |  | Reference |  |
| >1.4, <=1.8 | 0.65 (0.39, 1.07) | 0.093 | 0.98 (0.58, 1.64) | 0.935 | 0.91 (0.54, 1.54) | 0.730 |
| >1.8, <=2.6 | 0.81 (0.52, 1.27) | 0.362 | 1.82 (1.14, 2.91) | 0.013 | 1.58 (0.98, 2.56) | 0.063 |
| >2.6, <=3.4 | 1.04 (0.67, 1.61) | 0.874 | 2.43 (1.49, 3.96 | <0.001 | 1.99 (1.20, 3.29) | 0.007 |
| P for trend | <0.001 |  | <0.001 |  | <0.001 |  |

Model I was adjusted for Age, Gender, FPG, Cholesterol, Triglyceride, HDL-C.

Model II was adjusted for Age, Gender, BMI, SBP, DBP, FPG, Cholesterol, Triglyceride, HDL-C, Family history of diabetes.

HR, hazard ratios; CI, confidence interval; P-value, Probability value.
